# The two sides of 2D materials properties: a perspective on balancing air quality applications with environmental and health risks of 2D materials

**DOI:** 10.1039/d6va00035e

**Published:** 2026-05-08

**Authors:** Tse-Lun Chen, Yizhou Zhang, Ying Kong, Jing Wang

**Affiliations:** a Institute of Environmental Engineering, National Sun Yat-sen University Kaohsiung Taiwan; b Aerosol Science Research Center, National Sun Yat-sen University Kaohsiung Taiwan; c Advanced Institute for Materials Research (WPI-AIMR), Tohoku University Japan zhang.yizhou.e4@tohoku.ac.jp; d Institute of Environmental Engineering, ETH Zürich Zürich Switzerland jing.wang@ifu.baug.ethz.ch; e Laboratory for Building Energy Materials and Components, Empa Dübendorf Switzerland

## Abstract

Two-dimensional (2D) materials hold promise in revolutionizing air pollution control and airborne pathogen detection. However, their real-world application is entering a new phase that calls for a deeper understanding of their environmental and health risks. The field is now poised to bridge the gap between materials science and risk assessment. In this perspective, we present a vision that connects high-performance 2D materials applications with their unintended consequences. We discuss the life cycle impacts from manufacturing emissions to end-of-life concerns and the complex toxicological profiles of graphene-related materials and other emerging 2D materials families, and approaches to a “safety-and-sustainability-by-design” framework to guide the 2D materials research toward responsible, safe, and impactful societal solutions.

## Abstract 2

This perspective presents a “safety-and-sustainability-by-design” roadmap for 2D materials in air technologies, balancing high-performance applications such as sensing and purification with a deep understanding of life-cycle and toxicological risks.

## Graphical abstract

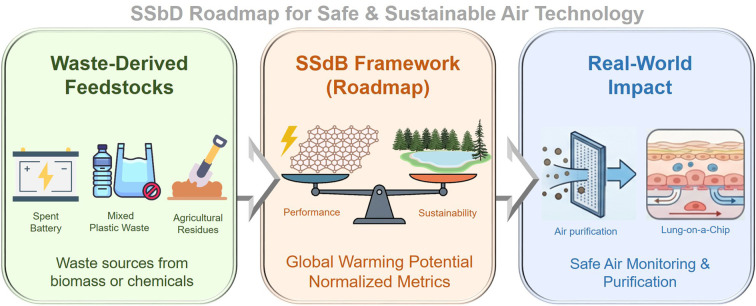

**Environmental significance**

While two-dimensional (2D) materials offer revolutionary potential for air quality monitoring and purification, their rapid development often overlooks associated environmental footprints and health risks, particularly regarding manufacturing emissions and end-of-life disposal. Addressing this dichotomy is crucial to prevent replacing one environmental hazard with another and to ensure sustainable real-world deployment. This work integrates material performance with life cycle assessment (LCA) and toxicological insights to propose a “safety-and-sustainability-by-design” framework. By advocating for responsible manufacturing and upcycling strategies, this perspective highlights the necessity of balancing technological efficiency with ecological safety, ensuring that emerging air quality solutions deliver genuine net benefits without introducing unintended environmental liabilities.

## 1 Introduction

Safeguarding air quality requires robust strategies that integrate effective purification and mitigation of airborne species with real-time sensing. Material platforms with engineered molecular interactions, transport, and optical characteristics are essential for converting trace contaminants into responsive signals, and enabling capture, degradation, or neutralization of harmful pollutants. Since the exfoliation of single-atomic-layer graphene in 2004, two-dimensional (2D) materials have attracted considerable interest for device-level applications in air pollution monitoring and purification.^1^ Advances in materials chemistry have expanded the family to include graphene derivatives *e.g.*, graphene, graphene oxide (GO), reduced GO, as well as transition metal carbides, carbonitrides, and nitrides (MXenes), phosphorene, layered double hydroxides (LDHs), and transition metal dichalcogenides (TMDs).^2–4^ Innovative bottom-up and top-down strategies now enable the atomic-level control of crystal structures and defects, providing a versatile platform for tailoring material functionalities.^1,5,6^ These unique physicochemical properties such as high surface-to-volume ratios and tunable surface chemistry have positioned 2D materials as transformative components in next-generation technologies.

Despite the success of 2D-material-based air technologies, the growing gap between laboratory-based innovation and real-world deployment now hinges on scalability, commercial viability, end-of-life disposal, and potential health hazards. Extensive research on 2D materials has sparked debates about their environmental risks and toxicological effects.^7^ One aspect linking proof-of-concept to practical applications requires systematic integration of life cycle assessment (LCA) and toxicological studies into materials design.^7^ Particularly in the context of sustainability, their integration into environmental and energy systems offers promising pathways for carbon capture, pollutant degradation, and advanced sensing, though their full lifecycle impact remains a critical consideration. Importantly, there is a largely underexplored design space for upcycled 2D materials and end-of-life materials, whose environmental and health implications must be properly understood and managed. During the production, processing, and disposal of graphene-related materials, for instance, airborne particulates may be released during manufacturing, to be inhaled and penetrate deep into the pulmonary region of the alveoli.^7^ The fabrication of MXenes typically requires chemically specific conditions for etching and surface functionalization.^8,9^ Meanwhile, upcycled 2D materials possess distinct performance and environmentally relevant advantages, as they inherently exhibit reduced lateral dimensions, increased defect density, and richly functionalized surface chemistry. While undesired for electronic or optoelectronic applications, deliberately harnessing heterogeneity through upcycling waste materials offers a pathway to transform recycled waste-derived materials into functionally optimized 2D materials platforms for enhanced sensing and purification, rather than being treated as environmental liabilities.

The deployment of upcycled 2D materials from waste remains constrained by both technological and institutional challenges, including device-level risk aversion and regulatory barriers. To bridge the next developmental gap, this perspective presents a viewpoint for integrating sustainability by design paradigm for air-related 2D materials applications. We first introduce life-cycle considerations, examine the feasibility of using various 2D materials to improve air quality, and establish a link between recycling-induced material properties and functional performance. We then discuss the toxicological profiles across major engineered 2D materials, to enable a more comprehensive connection of the widely varying material metrics and their potential health impacts. By integrating materials chemistry, sensing physics, purification mechanisms, and sustainability metrics, we aim to provide a forward-looking framework that promotes scalable, environmentally responsible, and high-performance air monitoring and remediation technologies based on 2D materials for the coming decade.

## 2 Life-cycle insight of 2D materials production and recycling exemplified by GRMs

The rapidly expanding family of 2D materials, led by graphene-related materials (GRMs), promises to revolutionize environmental monitoring and air-quality management industries. As this field matures, an opportunity emerges to develop a coherent, predictive framework for evaluating and guiding environmental sustainability.^13^ There exists a notable gap between current production capacity (9.7 kt per year) and actual demand (0.9 kt per year), which presents both risk and opportunity. The risk lies in the possibility that energy-intensive and environmental impacts become entrenched before full market maturity. On the contrary, this early stage offers an opportunity to steer the field toward sustainable synthesis, reuse, and recycling strategies before large-scale implementation.^13,14^

**Fig. 1:**
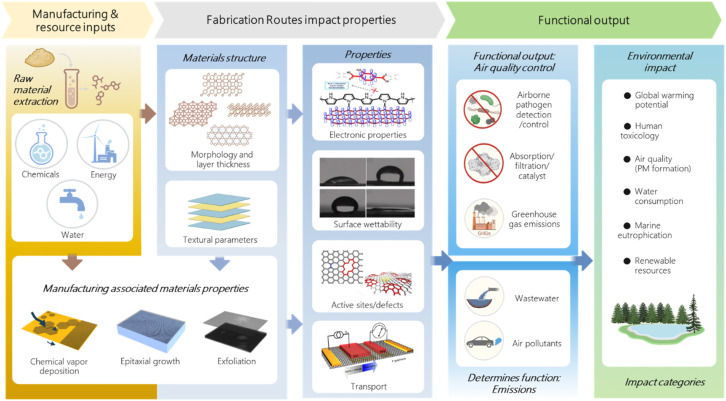
Schematic of the cradle-to-grave system boundary of GRM manufacturing associated with key materials properties. (Graphics representing textural parameters, surface hydrophilicity, and electronic properties were adopted with permission from ref. 10 and 11 Copyright 2015 and 2020 John Wiley & Sons, Inc. and from ref. 12 Copyright 2018 American Chemical Society).

We argue that the prevailing focus on materials performance, while necessary, can now evolve to incorporate more standardized LCA as a guiding design tool. The literature has documented a wealth of data on specific materials and processes on the environmental impacts of individual synthesis procedures. The next step is integrating these fragmented insights into a more predictive, application-oriented understanding of their environmental impacts. Fig. 1 shows the cradle-to-grave system boundary of graphene or GRMs when the manufacturing process is considered, and the application is limited to air pollution detection and control. No single synthesis pathway is universally optimal, so pristine GRMs are specifically tailored for polymer reinforcement, organic transistors, or filtration membranes by five dominant processes: (i) chemical reduction of graphite oxide, (ii) ultrasonication exfoliation, (iii) thermal exfoliation, (iv) chemical-vapor deposition (CVD), and (v) epitaxial growth. This diversity in synthesis allows for tailored material properties, which also complicates standardized environmental assessment. From the solvent-heavy chemical reduction of graphite oxide to the energy-intensive CVD, each pathway exhibits a unique environmental profile that has often been secondary to performance in lab-based studies. LCA helps to capture diversity using system boundaries from cradle-to-gate to cradle-to-grave, and determines the necessary 2D materials that are worth recycling. It is possible to account for upcycled materials and applications, including bulk exfoliation, etching, and device reuse. In particular, two methodological challenges emerge: synthesis diversity and the evolution of comparison metrics.

### 2.1 A barrier to standardized LCA of 2D materials

A comprehensive evaluation of the environmental impacts of 2D materials is based on the ISO 14040 standardized framework. Most LCA studies focus on the system boundary of graphene and GRM production, evaluating potential environmental impacts on a gate-to-gate, cradle-to-gate, or cradle-to-grave basis in four steps: goal and scope, inventory, impact assessment, and interpretation. However, LCA is data-intensive and demands substantial expertise, transparent analysis, and methodological consistency.^15^ Challenges such as multifunctionality, functional-unit choice, and system-boundary definition further complicate interpretation. Additionally, the use of different impact assessment methodologies, including CML, ReCiPe, TRACI, and ILCD, introduces further inconsistencies due to variations in indicators and normalization schemes. Overall, despite the promise of GRMs for scalable and potentially low-cost production, environmental impacts associated with the synthesis, processing, and disposal of pristine materials are often underemphasized. Several approaches have investigated eco-friendly reagents and processes, focusing on “safe and sustainable-by-design” (SSbD) methods to minimize harmful chemicals and harsh conditions.^14,16,17^ To transition beyond fragmented environmental data, the SSbD paradigm must be integrated into the earliest stages of 2D material synthesis. It dictates that hazard reduction (safety) and resource efficiency (sustainability) are treated as primary design objectives alongside material performance. In the context of 2D materials, this involves choosing non-toxic exfoliants, implementing closed-loop solvent recovery, and pre-emptively engineering material surfaces to minimize biological reactivity before they reach the application phase.

In one example of SSbD, upcycling waste materials containing graphite precursors represents a promising avenue for GRMs production when considering application-relevant sustainability metrics, typically involving thermal transformation and exfoliation of chemically complex feedstocks.^18,19^ The feasibility of the upcycling approach is supported by several successful implementations where waste-derived precursors were transformed into high-performance 2D platforms.^20^ For instance, flash Joule heating has been employed to convert diverse carbon sources ranging from mixed plastic waste to rubber tires into high-quality graphene with a significantly lower cumulative energy demand than traditional CVD methods.^21^ In the context of air quality, biomass-derived 2D carbon sheets synthesized from agricultural residues have demonstrated CO_2_ adsorption capacities and VOC removal efficiencies comparable to, or even exceeding, those of pristine graphite-derived GO, attributed to their inherent heteroatom doping and hierarchical porosity. Furthermore, the upcycling of silicon wafer waste and spent lithium-ion battery graphite into 2D nanosheets provides a dual benefit: mitigating hazardous waste while creating defect-rich surfaces that serve as ideal anchors for single-atom catalysts (SACs) in pollutant degradation. These examples underscore that upcycled 2D materials can meet the rigorous functional demands of air monitoring and purification while adhering to the SSbD paradigm.^22^ The carbon sources for GRMs include biological waste (*e.g.*, plant/animal-derived biomass, food waste, monosaccharides, animal-derived) and non-biological waste (*e.g.*, batteries, graphite, coal, oil, plastics).^18,23–34^ A critical distinction must be made between recycled and upcycled 2D materials within the LCA framework. While recycling typically refers to the recovery of materials for reuse in similar or lower-grade applications to reduce waste, upcycling involves the chemical or structural transformation of waste-derived feedstocks into 2D platforms with enhanced or specialized functionalities. From an LCA perspective, upcycling provides a ‘sustainability credit’ by diverting complex waste such as biomass or plastics and converting them into high-value sensing or purification agents, effectively lowering the cumulative energy demand compared to pristine synthesis from virgin graphite. With waste-derived sources considered, careful evaluation of product quality with respect to heteroatom doping, defects, layer count, lateral size, and defect is necessary.^13^ While producing high-quality pristine graphene for electronics from waste remains challenging, engineering recycled 2D materials by design is often advantageous for sensing and purification, especially when accounting for functionality-dominated sensing, adsorption, and catalytic conversion systems.^13,35–37^ On the one hand, the inherent heterogeneity of waste feedstocks often leads to batch-to-batch variations in layer count and surface chemistry. Maintaining industrial-grade quality control requires advanced sorting and monitoring technologies that are currently less mature than those for virgin materials. On the other hand, decentralized waste collection and the need for energy-intensive purification to remove contaminants can partially offset the sustainable credit of upcycling. Overcoming these technological and logistical barriers through standardized protocols will be essential for transitioning these platforms from laboratory proofs-of-concept to viable commercial air purification solutions.

### 2.2 The urgent need for consistent functional units

We envision a paradigm that expands the source of 2D materials for devices to include precursors upcycled from waste-derived feedstocks. Current GRMs major feedstock spans from graphitic and non-graphitic sources, as well as fossil-based sources, including biomass-derived precursors. A fundamental barrier to selection of feedstocks, synthesis routes, and end-user justification by meaningful LCA comparison, however, is the inconsistent choice of functional units. For example, inconsistencies arise when defining impacts per 1 kg of graphene produced *versus* per 1 cm^2^ of surface area for epitaxial growth. In pristine silicon wafer production, electricity use is a dominant contributor to the environmental impact of epitaxial graphene, and there exists significant potential for impact reduction through low-carbon energy sources or recycling. Sensitivity analysis emphasizes the importance of solvent recovery in ultrasonication exfoliation and chemical reduction routes.

The impact-benefit ratio (IBR) method has been employed to evaluate lifecycle trade-offs, where an IBR less than 1 indicates downstream benefits surpass upstream impacts. This approach, often evaluated using disability-adjusted life years (DALYs) and ReCiPe endpoint indicators, provides a useful guide to GRMs development for net environmental, health, or societal benefits.^38–40^ Global warming potential (GWP) is one of the most widely used LCA indicators for greenhouse gas emissions. For example, Cossutta *et al.* reported a GWP of 0.107 kg CO_2_ eq. per kg graphene for the Brodie method and 0.11 kg CO_2_ eq. per kg for the Staudenmaier method.^41^ Differences in GWP arise from the choice of oxidative agents, with potassium permanganate 1.78 kg CO_2_ eq. per kg, sodium chlorate 4.96 kg CO_2_ eq. per kg, and potassium chlorate ∼7 kg CO_2_ eq. per kg.^42^ Reported GWP results further depend on GRM structure and application, ranging from 146 to 586 kg CO_2_ eq. per kg for thin-film and nanoplatelet production.^42^ In contrast, methane-derived graphene has been associated with significantly higher GWP values of 36 000 kg CO_2_ eq. per kg and 82 800 kg CO_2_ eq. per kg, dominated by the contributions of natural gas combustion and hydrogen use.^43,44^ Therefore, we prospect that a direct comparison of these environmental footprints underscores the substantial sustainability gains of upcycling. Virgin 2D materials produced *via* conventional top-down (*e.g.*, chemical exfoliation) or bottom-up (*e.g.*, CVD) routes are inherently energy- and chemical-intensive due to high temperature requirements and specialized precursors. However, upcycling strategies, such as flash Joule heating or thermal conversion of biomass, bypass the environmental costs associated with raw material extraction and refined precursor synthesis.^21^ On the other hand, by utilizing waste-feedstocks which are assigned a zero-burden or even a sustainability credit in circular economy models, the cumulative energy demand can be reduced significantly compared to original production.

Collectively, GRMs production can be substantially less environmentally impactful when using natural or waste-derived feedstocks. However, the lack of consistent functional units and standardized protocols continue to impede reliable comparison across materials, processes, and applications. Addressing this gap is particularly critical for upcycled 2D materials intended for their applications, where surface exposure, defect density, and functional lifetime are often more relevant metrics than simple mass. To provide clearer guidance for future LCAs, we propose a transition toward performance-normalized functional units, such as the environmental impact per volume of air treated (kg CO_2_ eq. per 1000 m^3^) or per unit mass of pollutant removed.^41^ For instance, units based on the limit of detection or sensitivity per unit environmental cost would offer a more balanced justification for feedstock selection for sensing applications. To sum up, by incorporating functional lifetime and regeneration cycles into these metrics, it can more accurately quantify the net environmental gain of upcycled 2D materials compared to their pristine counterparts.

## 3 Advanced 2D materials properties for enhancing air quality

A critical distinction in the application-driven design of 2D materials lies in the contrasting requirements for structural perfection. While the semiconductor and optoelectronics industries prioritize pristine, large-area 2D materials with minimal lattice disruptions to ensure high carrier mobility, the requirements for environmental remediation are fundamentally different. Upcycled 2D materials, typically derived from waste feedstocks, often exhibit higher defect densities, reduced lateral dimensions, and richer surface functionalization compared to their pristine counterparts. Although these features are traditionally viewed as liabilities in electronics, they serve as functional assets in air quality applications. Specifically, the increased edge exposure and density of structural defects provide a vast library of active sites that modulate the binding energy for gaseous pollutants and promote interfacial interactions with particulate matter. By shifting the focus from crystalline perfection to defect engineering through upcycling, researchers can transform heterogeneous waste-derived platforms into high-performance, cost-effective agents for sensing, adsorption, and catalytic degradation.

To facilitate a more systematic comparison across different material platforms, we have summarized the life-cycle impacts, toxicological profiles, and application potentials of representative 2D material families in Table 1. This comparative framework reflects the strategic importance of balancing material performance with environmental and health safety, highlighting how upcycled forms can bridge the gap between sustainability and functional efficacy.

**Table 1:** Comparative overview of representive 2D material families for air quality applications

2D material family	Synthesis pathways & LCA insights (GWP)	Primary toxicological mechanisms	Potential in air quality applications	Advantages of upcycled/defect-rich forms
GRMs (graphene-related materials)	Pristine (CVD): 36 000–82 800 kg CO_2_ eq. per kg.	Induction of ROS, lung inflammation, and physical membrane disruption.^45,46^	PM filtration, VOC/toxic gas adsorption (NO_2_, NH_3_), and FET biosensing.^47–52^	Increased edge exposure and structural defects enhance gas binding energy and PM trapping sites.
	Upcycled: 0.1–0.11 kg CO_2_ eq. per kg.^43,44^			
MXenes	Requires chemical etching (HF/HCl–LiF); environmental burden linked to specialized precursors.^7^	Potential anti-inflammatory effects, but limited long-term (eco)toxicological data.^53–55^	Ultra-sensitive gaseous pollutant sensing and monitoring shielding applications.^56–58^	Surface terminations (–OH, –F) and vacancies provide diverse adsorption sites for polar molecules.^8,9^
LDHs (layered double hydroxides)	Generally lower energy demand routes; specific LCA data remains less explored.	Generally lower acute cytotoxicity; biotransformation concerns remain under-researched.^59^	Catalytic platforms for pollutant degradation and filtration additives.^60^	High ion-exchange capacity and tailorable interlayer spacing facilitate selective capture^57^
TMDs (*e.g.*, MoS_2_)	Exfoliation-heavy; LCA varies significantly by the identity of the chalcogen atom.	Chalcogen-dependent cytotoxicity; oxidative stress induction.^59^	Photoluminescence sensing and single-atom catalyst (SAC) supports.^61^	Structural defects (*e.g.*, sulfur vacancies) create unsaturated sites that maximize catalytic rates^3^

### 3.1 Balancing advanced air purification by monitoring and control

The structural and chemical diversity among 2D materials presents an intriguing opportunity to tailor devices based on specific air sensing and management demands. Outdoor and indoor air environments are exposed to various toxins, including airborne pathogens, fine particles, and harmful gases such as O_3_, CO_*x*_, NO_*x*_, SO_*x*_, and other volatile organic compounds (VOCs).^62,63^ Their concentrations and compositions vary geographically and are human-activity dependent.^64,65^ Conventional filtration units frequently struggle to capture the most penetrating particle sizes (<0.3 µm) without a significant pressure drop. 2D materials offer a pathway to overcome this limitation through tunable surface chemistry, high surface area, and various interfacial interactions. Importantly, many of these advantageous characteristics are preserved, or even enhanced, in upcycled and reclaimed 2D materials, which inherently possess higher defect densities, edge exposures, and chemical functionalities.

By integrating materials like graphene oxide (GO), MXenes, or layered double hydroxides (LDHs) as coatings, fillers, or functional additives, researchers have demonstrated enhancements in filtration efficiency for fine particulates and bioaerosols.^56–58^ Similarly, the high surface area and tunable chemistry of 2D materials have enabled impressive laboratory performance in adsorbing gaseous pollutants like NO_*x*_, SO_*x*_, and VOCs. Many of these performance metrics are reported in isolation from considerations of material sourcing, recyclability, long-term stability, and potential secondary contamination. In this section, we discuss the potential merit of upcycled 2D materials to provide a unique opportunity, with potential to combine high performance with sustainability, provided that their properties are matched to air sensing and purification functions rather than perfection in electronic uses.^66,67^

#### 3.1.1 Particulate matter filtration

2D materials have emerged as functional additives for air purification due to their straightforward integration of high-polarity surface groups within fibrous filtration systems. When blended or coated onto porous networks, these materials introduce strong surface interactions with particulate matter through enhanced dipole–dipole, induced-dipole, and electrostatic interactions within a three-dimensional nanoporous filter matrix. Coatings of GO, LDH, and MXene, can remarkably enhance air filtration performance and the overall quality factor.^47–52^

Blending 2D materials in nanocomposites is particularly attractive for particulate filtration, as single-defined crystallinity and lateral dimensions are not required for effective performance. For example, an ultrathin composite of GO and polydopamine applied to polypropylene filters refines the purification efficiency with minimal deviations in pressure drop.^68^ Similar benefits have been demonstrated using conductive 2D materials, where ion-driven alignment of reduced GO nanoplatelets on porous foam amplifies adsorption *via* electrostatic precipitation as a low-voltage ionizer on copper meshes.^69^ The assembly of charged graphene-based precursors within porous aerogels yields a robust, regenerable porous architecture that effectively traps particles.^70,71^ Within these systems, which leverage defect-rich, partially oxidized graphene, upcycled materials can offer unexplored opportunities, rather than pristine materials, by providing increased charge-trapping sites and interfacial roughness. Nevertheless, the use of 2D materials as a filter raises concerns about the impact associated with secondary pollution on material degradation and the release of nanoscale fragments during use or disposal. Addressing these challenges requires recycling-aware filter design strategies with proper immobilization and careful macroscopic structuring design.

#### 3.1.2 Gaseous pollutant removal: the challenge of regeneration and selectivity

**Fig. 2:**
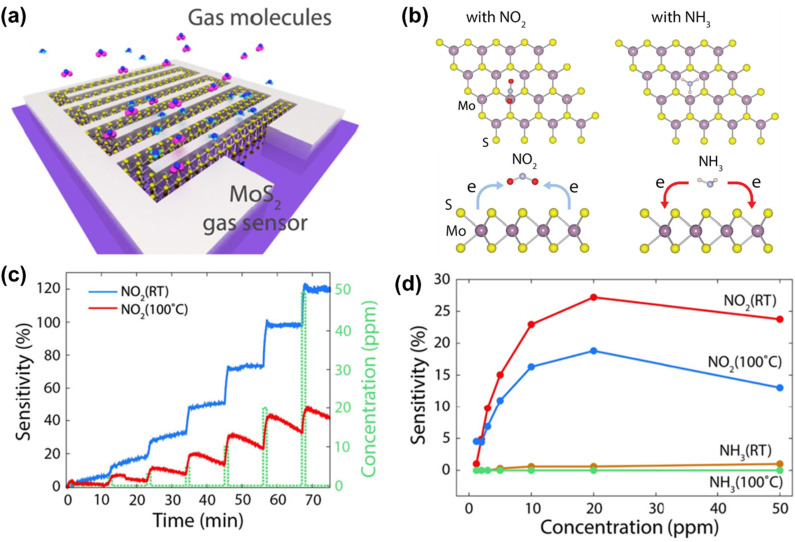
(a) 3D Schematic of the MoS_2_ gas sensor for NO_2_ and NH_3_. (b) Top views of preferred NO_2_ and NH_3_ adsorption on MoS_2_; charge-density difference plots for NO_2_ and NH_3_. (c) NO_2_ response *vs.* time (1.5–50 ppm) at RT and 100 °C; resistance increases for NO_2_, with faster recovery at 100 °C. (d) NO_2_*vs.* NH_3_ sensitivity across concentrations and temperatures; peak NO_2_ selectivity (NO_2_/NH_3_) at 20 ppm and 100 °C. Reprinted with permission from ref. 87, Copyright 2015, Nature.

Beyond filtration, 2D materials excel at selectively capturing toxic gases. In the case of GO, the carbonyl, hydroxyl, and carboxyl functional groups facilitate optimal gas molecule binding capacities through chemical reactions or tailored binding interactions. Notably, these materials have efficient adsorption capacities toward gases like NH_3_,^72,73^ NO_2_,^74,75^ SO_*x*_,^76–78^ H_2_S,^79^ and formaldehyde.^80^ The high performance in precisely distinguishing gas molecules for adsorption, such as sieving between water and toxicant gases, is particularly attractive for chemical vapor discharge.^81^ 2D materials from waste-materials precursor offer functional advantages to deliver diverse structures and surface functionalization to create a library of adsorption sites for tailored selectivity and binding strength. Functional groups and the exposed edges have been shown to bind with and identify different gas molecules.^82,83^ Hybrid systems such as LDH-GO composites,^84^ Ti-graphene systems,^85^ and Ca-decorated graphene have demonstrated the tunability of gas–surface interactions.^86^ For instance, the charge density differences for electron redistribution on MoS_2_ upon exposure to NO_2_ and NH_3_ enable electronic gas sensing (Fig. 2a–d).^87^

A central challenge in gas adsorption remains improved regeneration and long-term reuse. Adsorbed gas molecules can often be released through reduced pressure or inert gas substitution by flushing, though many materials demonstrate reduced capacity over time. The construction of three-dimensional macrostructures was proposed for more straightforward and economical regeneration,^88^ as they mitigate the need for precise nanoscale control while maximizing accessible surface area. Recent studies have also explored the use of GO-based systems for CO_2_ capture and reduction in flue-gas environments, especially when combined with electrochemical or photocatalytic functionalities.^78,89–91^ In these applications, upcycled 2D materials may serve dual functions, both as adsorbents and active catalytic or charge-transfer components.

#### 3.1.3 Catalysis and degradation: the frontier of single-atom catalysts

Perhaps the most advanced frontier in air-relevant purification is the use of 2D materials as platforms for SACs, designed to degrade pollutants rather than passively capture them. Confined single atoms in 2D structures tend to be more coordinatively unsaturated, providing full exposure of catalytic sites to reactants for maximized rate of reactions, resulting in new electronic states and thereby enhancing the performance as a heterogeneous catalyst. In the past ten years, a broad range of graphene-like 2D materials have been synthesized, ranging from hexagonal boron nitride (h-BN) and graphitic carbon nitride (g-C_3_N_4_) to TMDs, LDHs, 2D metals, 2D zeolites, and 2D metal–organic frameworks (MOFs). 2D materials have served as standards for investigating genuine active sites and surface reaction mechanisms by combining theoretical calculations with surface science. Furthermore, SACs have been developed on various 2D supports, including C, MgO, Al_2_O_3_, SiO_2_, TiO_2_, FeO_*x*_, ZnO, CeO_2_, and MoC, for thermocatalytic, electrocatalytic, and photocatalytic applications. The concept of 2D materials confined SACs involves confining targeted electronic structures through doping or anchoring atomic clusters on the host structure to modulate the coordination environment.^60^

Research needs to reliably scale up the 2D materials SACs from laboratory output with robust performance and economic viability, and the selectivity to remove target pollutants. Beyond manufacturing pristine 2D materials systems, upcycled and defect-rich 2D materials offer particularly favorable environments for single-atom anchoring and present a practical pathway toward scalable and selective catalytic platforms. To understand their properties, accurate probing of atomic structure and electronic states, facilitated by advanced characterization techniques and theoretical calculations, can guide predictions of molecular reaction dynamics.^60,92,93^

### 3.2 Can upcycled 2D materials efficiently and precisely detect airborne pathogens?

The COVID-19 pandemic highlighted the urgent need for technologies capable of rapidly detecting and neutralizing airborne pathogens.^94^ 2D materials have emerged as a powerful multifunctional platform, offering a dual role as the active element in ultra-sensitive biosensors for on-site detection, and as functional materials for the active capture and inactivation of viruses and bacteria.^95–99^ These interactions are further enhanced by topological characteristics, the potential for functionalization, achieved through covalent modification of surface chemical handles, direct chemical doping, ligand exchange, π-complex formation, selective counterion immobilization, and the deposition of nanoparticles.^100–103^ Different sources of 2D materials can offer surface features essential for biosensing, enabling the biofunctionalization for the attachment of biomolecules such as DNA, enzymes, and proteins.^104–108^ While the literature is replete with proof-of-concept demonstrations with pristine materials, a comprehensive evaluation of practical viability, safety, robustness, biosafety, and long-term performance, particularly for the prospective reclaimed 2D materials, is required towards the development of cost-effective public health tools.

#### 3.2.1 Ultrasensitive detection with LCA consideration: the quest for real-world robustness

**Fig. 3:**
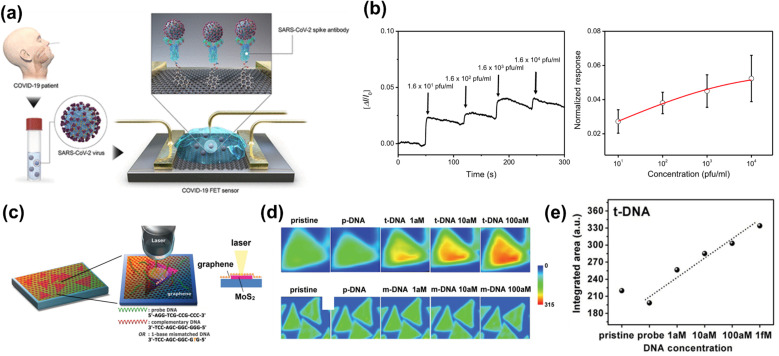
(a) COVID-19 FET using graphene with spike antibodies linked by 1-pyrenebutyric acid NHS ester. (b) Real-time FET response and dose-response to cultured SARS-CoV-2. (c) DNA detection scheme with graphene/MoS_2_ sensor and optical readout. (d) Photoluminescence maps for target DNA and a mismatch. (e) Integrated photoluminescence signal for target DNA at 1–1000 aM. (a) and (b) are reprinted with permission from ref. 110, Copyright 2020, American Chemical Society. (c)–(e) are reprinted with permission from ref. 115, Copyright 2014, John Wiley & Sons, Inc.

The exceptional electronic and optical properties of 2D materials are useful in biosensing.^99,109,110^ For example, by functionalizing graphene sheets with antibodies that target the SARS-CoV-2 spike protein, the change in electrical properties upon binding can be evaluated as a field-effect transistor (FET) biosensor, which measures the current–voltage characteristics of binding with a limit of detection of fg L^−1^ in both culture medium and clinical samples (Fig. 3a–b).^110^ Thionin-functionalized molybdenum disulfide (MoS_2_) has been demonstrated to act as an efficient electrochemical sensor for both single-stranded DNA (ssDNA) and double-stranded DNA (dsDNA) by cyclic voltammetry.^104^ The sensing by differential pulse voltammetry is also possible with MoS_2,_ with an atto-molar level limit of detection.^111^ Anchoring gold nanoparticles on tungsten sulfide-graphene (WS_2_-Gr) composites amplifies the electrochemical signal in differential pulse voltammetry, enabling it to discern between single-base mismatches and three-base mismatches in DNA sequences with a limit of detection of 2.3 fM.^112,113^ However, this approach faces implementation challenges on a large scale because of the high electrochemical standard that requires pristine 2D materials with well-controlled surface properties. The early success in the applications of upcycled 2D materials on electrochemical sensing is more likely for qualitative detection of trace analytes.

Optical biosensing strategies benefit from the carrier and signal-modulation capabilities of 2D materials. Tailored surface binding allows for multiplex, rapid, selective, and sensitive detection of biomolecular targets in homogeneous solutions. Taking the advantage of high fluorescence quenching efficiency, graphene-based multicolor fluorescent DNA devices selectively hybridize the target DNA of the associated dye fluorescence and integrate multiple detection capabilities within a single probe.^61^ This capability also enables enzyme-free, *in situ* miRNA imaging and quantification by catalyzed hairpin assembly based on GO carrier.^114^ Synthesis of a hemin-graphene hybrid nanosheet functionalized by π–π interactions coupled the merits of both hemin and graphene, and this character is combined with different affinities of the composite towards single-nucleotide polymorphisms with label-free colorimetric detection.^114^ Another label-free sensor function by photoluminescence detection is prepared by stacking graphene on grown MoS_2_ into a heterostructure. The graphene protects the stable MoS_2_ structure and provides a handle for hosting DNA molecules with a limit of detection down to 1 aM level (Fig. 3c–e).^115^ Upcycled materials may better maintain the optical sensing performance while offering improved manufacturability. Nonetheless, achieving real-world robustness requires shifting emphasis from pristine material quality toward controlled surface chemistry, mechanical robustness, and lifecycle-aware precursor synthesis.

#### 3.2.2 Active capture and inactivation by 2D materials

**Fig. 4:**
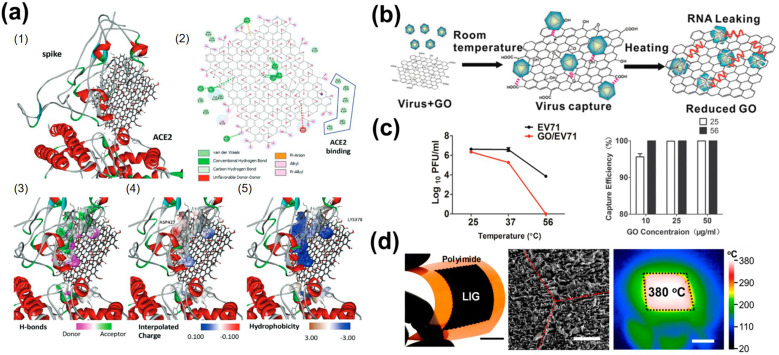
(a) GO-6M0J docking analysis: (1) docking pose (binding −9.1 kcal mol^−1^); (2) GO-6M0J interaction map (spike residues listed; ACE2 residues listed); (3) hydrogen bonds (donors pink, acceptors green); (4) ASP427 charge interactions; (5) hydrophobic/π-contacts (LYS378). (b) Temperature-dependent GO-virus interactions with dotted lines for physiochemical contacts; RNA release shown in red. (c) Temperature- and GO-concentration-dependent EV71 capture and GO/EV71 removal efficiency. (d) Optical image of a flexible LIG filter (4.5 × 4.5 cm2) with a 2 cm scale bar, SEM view of the LIG carpet with unlased PI (red dashed line, 500 µm), and IR image of a 380 °C heated LIG filter (black outline). (a) Is reprinted with permission from ref. 119, Copyright 2021, John Wiley & Sons, Inc. (b) and (c) are reprinted with permission from ref. 120, Copyright 2015, John Wiley & Sons, Inc. (e) Is reprinted with permission from ref. 21, Copyright 2019, American Chemical Society.

Beyond molecular sensing, 2D materials also function as robust platforms for the capture, inactivation, and removal of airborne pathogens through tailored surface interactions. This approach is promising because 2D materials enable efficient bioaerosol collection at high flow rates, while also facilitating downstream pathogen identification through RNA or DNA analysis. Importantly, the pristine properties with crystalline perfection are less important, but the surface area, flexibility, and diverse surface chemistries on upcycled 2D materials provide functionalities to their applications. GO has already demonstrated label-free interactions with biological species, enabling the capture and denaturation through a combination of electrostatic attraction, hydrogen bonding, and pi–pi interactions across multiple length scales.^116,117^ For example, grafting sulfate mimetic polyglycerol sulfate onto GO enables the adsorption of heparan sulfate-dependent virus with their positively charged residues, hence blocking its entry and replication.^118^ GO nanosheets were also shown to directly interact with SARS-CoV-2 spike proteins and interfere with cell receptors to disrupt their infectivity, even in the presence of mutations on the viral spike (Fig. 4a).^119^ These interactions also facilitate viral RNA extraction through facile bioreduction (Fig. 4b–c).^120^

The ability of 2D materials in pathogen control is further expanded by their ability to undergo stimulus-responsive sterilization.^121^ Such capability is relevant to direct virus disruption and can be augmented with additional functionalities, including laser-induced self-sterilizing graphene. The photothermal conversion of waste polyimide film by a CO_2_ laser cutter enables the direct construction of macroscopic architectures, and the tailored periodic Joule heating above 300 °C can efficiently destroy adhered microorganisms (Fig. 4d).^21^ A similar strategy employed a graphene-tungsten oxide composite that is responsible for visible light irradiation for photoinactivation, leading to disruption of viral protein capsid and the release of RNA within the viral envelope.^122^ While other 2D materials, such as MoS_2_ and graphitic carbon nitride, have been reported to exhibit antimicrobial activity, further investigation is required to determine their potential for efficiently combining microbial capture and disinfection capability.^123^ In this context, upcycled 2D materials offer a compelling opportunity to develop scalable, multifunctional air-treatment systems to enable efficient pathogen interception and disinfection.

## 4 New paradigms in 2D material toxicology

The rapid proliferation of 2D materials across manufacturing, upcycling, and consumer sectors necessitates a robust understanding of their human health risks, particularly *via* inhalation during processing or disposal.^59^ However, establishing universal toxicological guidelines remains a formidable challenge, as subtle variations in material properties often overlooked in standardized screenings can lead to disproportionately large discrepancies in biological outcomes. This structural and functional complexity, coupled with the dynamic nature of the bio-interface, has led to a serious impasse of inconsistent and often contradictory findings. The emerging topic of 2D material health concerns the potential adverse interactions of these materials at the nano-bio interface. To determine the biological interactions and fate of 2D materials *in vivo*, their chemical composition is one of the most important considerations. We envision that these divergent impacts arise not only from the inherent complexity of 2D platforms but also from the limitations of traditional toxicological models. Consequently, the field must transition toward evaluating how specific material properties drive biological responses within physiologically relevant and dynamic environments.

### 4.1 The limitations of correlating material properties to inconsistent outcomes

Research so far has diligently characterized the physicochemical factors influencing the biological fate of 2D materials. Properties such as chemical composition, lateral size, thickness, and surface functionalization are known to govern biodistribution and toxicity.^59^ Initial studies comparing the cytotoxicity of various TMDs have shown that the identity of the chalcogen atom plays a crucial role in overall cytotoxicity, likely due to differences in chemical reactivity. Cytotoxicity is often used as a metric to assess the biocompatibility of emerging 2D materials, given the relatively limited toxicology data available for these materials. The exfoliation of layered materials changes their fundamental properties, and biological interactions with layered materials vary with exfoliation state.^124,125^ Inorganic materials generally degrade less readily than organic substances, raising concerns about potential health and environmental impacts during processing and mechanical treatments. Mechanical processing, abrasion, or improper disposal of products like graphene-enhanced filters can release airborne particles, which may be inhaled and reach the alveoli, interacting with lung tissue. Besides GRMs, similar behaviors have been observed with other carbon-based materials, such as carbon black and carbon nanotubes.^126,127^ The flexible, planar structure of 2D materials allows morphological reconfiguration, affecting interactions with biological barriers and leading to unique biodistribution and metabolism compared to traditional organic carriers, often complicating their biological behavior.^128^

Studies on the degradation and metabolism of graphene and GRMs in mammals indicate that biosafety is influenced by multiple factors. Size critically affects cellular uptake, clearance, biodistribution, and blood–brain barrier penetration, while surface chemistry governs ion and protein adsorption, impacting circulation time. In mice, high doses of GO (10 mg kg^−1^) did not cause histopathologic damage, and most GO was excreted *via* the kidneys within a month, becoming undetectable after 240 days.^128–131^ Reduced GO, however, accumulated in organs such as the liver, spleen, and kidney, leading to hepatotoxicity and immune responses due to slow clearance.^132^ In addition, toxicity studies on 2D materials have primarily focused on their dispersions in structural forms, as these are most common in biological applications like drug delivery and imaging. Increasing interest lies in exploring other forms, such as thin films, 3D constructs, and composites. Surface functionalization significantly influences their toxicological responses, as it defines their biomolecular identity. For example, biomolecular coronas formed by capping GRMs with amino acids or blood proteins can alter biodistribution, reduce hemolytic effects, modulate cell targeting, and regulate immune responses, therefore decreasing cytotoxicity.^133–135^ Coatings with antifouling agents like polyethylene glycol or dextran also diminish non-specific adsorption.^136^ Therefore, the biological response to individual 2D materials is uncertain, partly due to the lack of standardized nomenclature and classification systems,^137^ as well as limited understanding of how exposure routes including inhalation, ingestion, dermal contact, or injection affect outcomes.^138^ Factors such as lateral size, thickness, number of layers, functionalization, and aspect ratio influence bio-distribution and toxicity mechanisms. Smaller, thinner 2D materials with larger surface areas tend to adsorb more endotoxins, inducing inflammatory cytokines and increasing cytotoxicity. To better understand size-dependent effects, variables like endotoxin contamination in the process should be carefully controlled.^139,140^

### 4.2 Physiologically relevant *in vitro* models

**Fig. 5:**
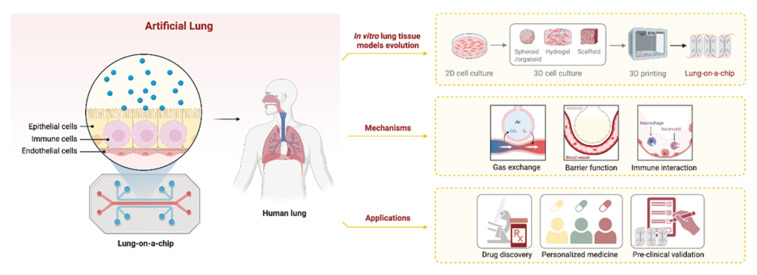
Schematic overview of the lung-on-a-chip as an advanced artificial lung model, encompassing its biomimetic design, the evolution of *in vitro* modeling techniques, recapitulated physiological mechanisms, and key biomedical applications.

The preclinical safety assessment of 2D materials has been hindered by inconsistent and often contradictory findings, creating a serious impasse in accurately determining their risk to human health. For instance, while GRMs may show low acute cytotoxicity in simple models of the gut and placenta, chronic exposure consistently reveals underlying issues like the induction of reactive oxygen species (ROS) and inflammation.^45,46^ These divergent findings arise in part from differences among material types. While GO is frequently reported to induce lung inflammation in several studies, graphene quantum dots and certain MXenes have demonstrated anti-inflammatory potential under different conditions or experimental setups.^53–55^ This heterogeneity underscores not only the complexity of the materials, but also limitations in the current models. There is an urgent need for more physiologically relevant, standardized, and reproducible platforms that can better predict human responses across a range of materials. The challenge of accurately predicting these complex biological responses is exacerbated by the limitations of traditional preclinical models. Animal studies, while informative, are costly, ethically challenging, and do not always reliably predict human-specific outcomes. Similarly, conventional 2D *in vitro* cell cultures fail to replicate the intricate architecture and dynamic environment of human organs like the lung, making it difficult to assess the true impact of inhaled nanomaterials.^141–143^ To bridge this gap, advanced *in vitro* platforms like the artificial lung system have emerged as a powerful solution, as shown in Fig. 5. These microphysiological systems are designed to mimic human lung structure and function with high fidelity, providing a more accurate and human-relevant platform for toxicological and therapeutic evaluation. A schematic of such a system, often referred to as a “Lung-on-a-Chip”. The technological evolution of lung tissue models, progressing from traditional 2D cell cultures to more complex 3D constructs (*e.g.*, spheroids, hydrogel scaffolds), 3D printing, and culminating in the organ-on-a-chip platform. It also highlights the physiologically relevant mechanisms that can be investigated using this model, including gas exchange, endothelial/epithelial barrier function, and immune cell interactions. Furthermore, by incorporating key features such as a dynamic air–liquid interface (ALI) for realistic aerosol exposure, simulated breathing motions through mechanical forces, complex co-cultures of multiple cell types including epithelial, endothelial, fibroblast, and immune cells and integrated real-time monitoring capabilities, these models are more versatile than simpler systems. Integrating these features enables a comprehensive assessment of key toxicity endpoints including oxidative stress, cytokine release, and barrier integrity under conditions that closely resemble human respiration. This offers a high-throughput, ethically sound platform that is more predictive of human responses to 2D materials in the lung. For instance, an optimized lung-on-a-chip system that achieves high epithelial barrier integrity under ALI and mechanical stretching conditions. The platform demonstrated 72% accuracy in predicting acute inhalation toxicity, proving its potential as a standardized, human-relevant alternative to animal testing.^144^ A latest study demonstrates that Ta_4_C_3_ MXene nanosheets, particularly the 100 to 500 nm size fraction, exhibit strong biocompatibility and multifunctional therapeutic potential including ROS scavenging, anti-inflammatory macrophage polarization, and antifibrotic effects when evaluated in an advanced immunocompetent 3D ALI lung triculture model.^145^ While the primary focus is on toxicity evaluation, the same advanced features also support exploration of therapeutic potential under carefully controlled conditions, hence accelerating preclinical validation, enabling larger-scale screening, and informing strategies for personalized medicine.^139,146–149^

### 4.3 Public perception and the social dimension of 2D material adoption

Beyond technical and safety milestones, the large scale adoption of 2D material-based air quality solutions such as household air purifiers or wearable sensors is heavily influenced by public perception. A primary barrier to acceptance is the consumer's concern regarding nanomaterial exposure, where the potential release of nano-sized fragments or nanoparticles during device aging, mechanical abrasion, or improper disposal. Based on the toxicological impasses suggests that even if laboratory-scale studies indicate low acute toxicity, the long-term biological impact of inhaled fragments such as ROS induction or chronic lung inflammation remains a significant public health question. To build public trust, it is insufficient to merely report high purification efficiency; researchers must prioritize the development of robust macroscopic architectures that ensure 2D materials are securely immobilized, thereby minimizing the risk of secondary airborne exposure throughout the product's functional lifetime.

Simultaneously, aligning 2D material innovation with the principles of a circular economy offers a pathway to enhance societal acceptance through resource efficiency. By integrating the SSbD framework, the field can transition from energy-intensive pristine synthesis to the responsible upcycling of waste feedstocks. As demonstrated by LCA results, upcycled 2D materials significantly reduce the GWP and cumulative energy demand compared to traditional manufacturing routes. This paradigm shift not only mitigates the environmental burden associated with raw material extraction but also transforms perceived environmental liabilities into high-value functional agents. Ultimately, demonstrating both the physical containment of nanomaterials and the sustainable lifecycle of waste-derived precursors will be critical for the successful market integration of 2D material technologies.

## 5 Conclusions

The deployment of 2D materials from laboratory results in societal practice is being hampered by a fractured understanding of their safety and environmental footprint. Contradictory toxicological data and inconsistent LCA stem from inherent limitations in our perspectives, including studying these materials in isolation, ignoring the dynamic transformations they undergo in real-world biological and environmental systems. Notably, limited (eco)toxicological data are available for novel advanced 2D materials such as MXenes and LDHs, underscoring the need to assess their potential health and environmental impacts. GRMs and other 2D materials typically undergo biotransformation, meaning it is insufficient to characterize the properties of as-produced 2D materials merely. Toxicologists should also consider the many transformations (*e.g.*, agglomeration, dissolution/degradation, coronation) that may occur in natural environments or within the human body, both extracellularly and intracellularly.

Overall, the integration of 2D materials in air applications should have a more thoughtful consideration not only on their performance, but also on the health and environmental impact. While the lifecycle of 2D materials production may impact carbon emissions due to energy and resource demands, the synergistic environmental benefits from pollution control or novel energy transformations should be re-evaluated. We envision that upcycled 2D materials can provide an alternative means for improved performance metrics, with more careful consideration given to the cost and environmental burden. Therefore, we propose a strategic SSbD roadmap to transit the 2D materials from laboratory research to societal practice. First, the field needs to adopt standardized, performance-normalized LCA metrics to accurately quantify the “sustainability credit” and reduced cumulative energy demand of upcycled waste-feedstocks. Second, material design should shift from pursuing crystalline perfection toward leveraging the functional advantages, upcycled 2D platforms such as enhanced edge exposure and surface functionalization specifically tailored for air purification and sensing. Third, preclinical safety assessments should evolve from static models toward physiologically relevant, dynamic platforms like the lung-on-a-chip to more closely capture human bio-interactions and respiratory stress. Finally, ensuring secure material immobilization within robust macroscopic architectures is essential to minimize secondary exposure risks and build the public trust necessary for market adoption. As a more comprehensive upcycled 2D materials library becomes available, and the metrics for toxicology under real environmental or physiological conditions become better established, continuing advances in their applications will help transition the 2D materials from laboratory to commercial products.

## Author contributions

T.L Chen, Y. Kong, and Y. Zhang conceptualized, wrote, and data cruising to the Perspective. Y. Zhang and J. Wang supervised, and edited of the manuscript.

## Conflicts of interest

There are no conflicts to declare.

## Data Availability

No primary research results, software or code have been included and no new data were generated or analysed as part of this review.
